# Patients’ willingness to pay for their drugs in primary care clinics in an urbanized setting in Malaysia: a guide on drug charges implementation

**DOI:** 10.1186/s12930-017-0035-5

**Published:** 2017-04-04

**Authors:** Sharifa Ezat Wan Puteh, Siti Nurul Akma Ahmad, Azimatun Noor Aizuddin, Ramli Zainal, Ruhaini Ismail

**Affiliations:** 1 Faculty of Medicine, Department of Community Health, University Kebangsaan Malaysia Medical Center, Jalan Yaacob Latif, Bandar Tun Razak, Cheras, 56000 Kuala Lumpur, Malaysia; 2 Health Administration, Faculty of Business Management, Universiti Teknologi MARA, Puncak Alam, Selangor Malaysia; 3 Institute for Health Systems Research, Ministry of Health, Kuala Lumpur, Malaysia; 4 Health District Office, Sepang, Malaysia

**Keywords:** Willingness-to-pay, drugs expenditure, Acute diseases, Chronic conditions, Urban state

## Abstract

**Background:**

Malaysia is an upper middle income country that provides subsidized healthcare to ensure universal coverage to its citizens. The challenge of escalating health care cost occurs in most countries, including Malaysia due to increase in disease prevalence, which induced an escalation in drug expenditure. In 2009, the Ministry of Health has allocated up to Malaysian Ringgit (MYR) 1.402 billion (approximately USD 390 million) on subsidised drugs. This study was conducted to measure patients’ willingness to pay (WTP) for treatment of chronic condition or acute illnesses, in an urbanized population.

**Methods:**

A cross-sectional study, through face-to-face interview was conducted in an urban state in 2012–2013. Systematic random sampling of 324 patients was selected from a list of patients attending ten public primary cares with Family Medicine Specialist service. Patients were asked using a bidding technique of maximum amount (in MYR) if they are WTP for chronic or acute illnesses.

**Results:**

Patients are mostly young, female, of lower education and lower income. A total of 234 respondents (72.2%) were not willing to pay for drug charges. WTP for drugs either for chronic or acute illness were at low at median of MYR10 per visit (USD 3.8). Bivariate analysis showed that lower numbers of dependent children (≤3), higher personal and household income are associated with WTP. Multivariate analysis showed only number of dependent children (≤3) as significant (*p* = 0.009; 95% CI 1.27–5.44) predictor to drugs’ WTP.

**Conclusion:**

The result indicates that primary care patients have low WTP for drugs, either for chronic condition or acute illness. Citizens are comfortable in the comfort zone whereby health services are highly subsidized through universal coverage. Hence, there is a resistance to pay for drugs.

## Background

The increase in drug expenditure is a crucial challenge in Malaysia. As there is no established compulsory National Health Insurance yet, most of the patients obtain treatment including prescription of drugs from government funded health clinics and hospitals [1]. The Ministry of Health delivers comprehensive medical, health, dental and pharmaceutical services at a subsidized rate leading to increase burden on the government. Since year 2000, the national drug expenditure has increased from MYR 346 million (USD 91 million) to MYR 915 million (USD 241 million) in 2005. From the year 2004–2005, an increase of 13.3% was recorded [2] and according to the Pharmaceutical Services Division Annual Report, the Malaysia Ministry of Health drug expenditure had escalated to MYR 1.402 billion in 2009 [3].

Numerous factors [2, 3] are found to contribute to an increase drug prices including growth of aging population, higher consumer expectations, long-term drug treatment for chronic conditions, polypharmacy, improvements in diagnostics/treatment of diseases, and novel expensive drugs offered due to advancement of health technology [4]. As a result, the government has an increasing demand on the rising health care costs [1, 3]. Provision for chronic conditions and acute diseases drugs take place in many primary care facilities throughout the state and yet, they are free of charge. A national health insurance has been proposed to be strategically implemented in the government funded primary care centers in Malaysia in the very near future [5]. Therefore, this study intends to measure patients’ WTP for their drugs who attend to public primary care services.

### Willingness to pay for drugs

Willingness to pay is a methodological tool to discover the hypothetical monetary value for programs and specific medical interventions and treatments [6]. A study that examined the WTP for Praziquantel treatment (for Schistosoma parasites) in Ogun State, Nigeria, showed 92.3% respondents were willing to pay for the drug to treat the infected household members [7]. However, in a coronary restenosis (re-narrowing) study that assessed patients’ WTP to avoid revascularization procedure, found that the proportion of patients’ WTP is higher with greater absolute risk reductions [8]. This is logical in the sense that higher risk would entail higher commitment and WTP. Similarly, in assessing WTP for cancer prevention, the results revealed that income and the probability of developing cancer were positively correlated to WTP [9].

Malaysia state healthcare system has been heavily financed through the government tax based financing, although its health care prosper under bipartite public and private providers. Under the universal health coverage, Malaysia’s public out patients healthcare is provided almost fee exempted and nominal payment upon admission as inpatient. Employer-based financing covers a limited number of companies and private insurance, that engage private health providers, mostly are concentrated in urban areas. Primary care facilities in Malaysia are highly subsidized by the government under the universal coverage, leading to very low state cost recovery. Malaysian citizens have to pay a user fee of only MYR1 (estimated to be USD 0.38) for each primary care outpatients’ payment. The course of drugs prescribed ranged between 5 days–1 week for acute illness and 1–3 months’ supply for chronic condition. Besides drugs; disease screening, investigations such as blood test and radiological imaging are highly subsidised and mostly done for free. In the future, a national health insurance system has been proposed to be implemented in the hope of reducing the cost burden of the government providers.

Many primary care clinics in Selangor are equipped with Family Medicine Specialist (FMS) and case-mix services (using the ICD-10 ambulatory coding and grouping). These facilities and logistics are placed due to the higher number of population seeking services, higher patients’ expectancy, an increasing aging population and the increasing severe and complex case mix cases.

## Methods

### Study population

This study is a cross-sectional study conducted in year 2012–2013 Selangor, Malaysia. The population represents both high and low income quintile level of population coming from both urban and rural areas. In each district, there are two to six public primary clinics, equipped with clinician with specialty trainings, for example the Family Medicine Specialists (FMS). A total of 10 public primary care with existing FMS services were chosen as those clinics are visited by patients who suffer from various types of diseases; managed with various types of medications including the “list A” drugs (i.e. for example drugs that can only be prescribed by specialists).

### Definition of “chronic condition” and “acute illness”

This study aims to determine patients’ WTP for drugs, both for chronic conditions or acute illnesses. Chronic condition is defined as conditions that had lasted or were expected to last 12 or more months and result in functional limitations and/or the need for on-going medical care [10]. The WTP among patients were examined with three most prevalent chronic conditions in Malaysia which were hypertension, type-2 diabetes mellitus and ischemic heart disease (IHD). Acute illness is defined as rapid onset and/or short course disease less than 3 months duration, which is self-limiting or requiring minimal treatment [11].

### Sample size and sampling method

The sample size was calculated by considering the assumption of two population proportion formula: the proportion (P1) = 1.2% of low income that are willing to pay, while proportion (P2) = 11% of high income that willing to pay [12]. The *Z *(standard normal) distribution value at 95% confidence level was taken at 1.96, 5% of absolute precision, and 20% non-response rate. Hence, the total sample size with consideration of study design effect was n = 324. Implementing two-stages random sampling, ten public primary cares with FMS service were selected through computer generated random sampling. Subsequently, 33 patients from each health clinic were designated via systematic random sampling. A total of 324 adults (aged 18 years and above) and of local residents were chosen as our respondents. Clinically or mentally unstable patients or with the case mix of severity level III (from either acute or chronic conditions) that were deemed too severe for the bidding interviews were excluded from the study.

### Study tools

The combination of modified questionnaire and validated self-developed questionnaire were used. The questionnaire consisted of four parts which are socio-demographic data, patient treatment expenses, patients’ WTP and the maximum monetary amount that each patient is willing to pay for drugs procurement.

### Data collection

Primary care clinics are perceived as to be more close-knitted with the local community, as compared to patients from hospitals that may receive referral from other states. Pretested and structured questionnaires through face-to-face interviews with 324 respondents were successfully carried out.

### Data analysis

Analysis was generated using SPSS version 20 software, and the p value of less than 0.05 was accepted as statistically significant. The relationships, proportion and control of confounders, respectively were conducted using the Mann–Whitney U test, Pearson Chi square test and patients’ WTP as the categorical dependent variable. The multivariate model was adjusted for demographic characteristics. Model fitting was assessed by the change in 2 log-likelihood value and the significance of random parameter variance estimate was assessed using the Wald test. The values will be presented in Malaysian Ringgit (MYR) currency which stands at an estimated conversion of MYR 3.8 to USD 1 (as of July 2015).

## Results

### Socio-demographic and characteristics of health condition of study participants

The total respondents of 324 showed the distribution of the patients’ age from the younger age group. A total of n = 169 (52.2%) respondents were from the younger age (18–47 years), while the rest were from older age group (≥48 years) (Table 1). The majority of respondents at n = 116 respondents (35.8%) were working with the private sector. Half of the respondents were from the lower personal income level (MYR 0–950/month) and low household income (MYR 0–1900/month) group. Only 103 (31.8%) of respondents had any form of private voluntary health insurance with an average premium payment of MYR110 per month. Only 28 respondents (8.6%) had ever accessed private hospital treatment. All respondents visit the subsidised public health care extensively, while only 54.9% of patients had ever been admitted to public hospitals. 50% of patients also regularly visit private health clinics for their healthcare services.

**Table 1:** Frequency distribution of patients’ socio-demographic

Variables	Frequency (n = 324)	%
Age (years)		
Younger (18–47)	169	52.2
Older (48 and above)	155	47.8
Gender		
Male	146	45.1
Female	178	54.9
Ethnicity		
Malay	192	59.3
Chinese	30	9.3
Indian	95	29.3
Others	7	2.2
Marital status		
Married	256	79.0
Single	52	16.1
Widow	16	4.9
Level of education		
Lower level of education	246	75.9
Higher level of education	78	24.1
Personal income		
Lower income (RM0-950)	163	50.3
Higher income (RM951 and above)	161	49.7
Household income		
Lower income (RM0-1900)	165	50.9
Higher income (RM1901 and above)	159	49.1
Disease status		
Chronic conditions	162	50.0
Acute illness	162	50.0
Insurance status		
Yes	103	31.8
No	221	68.2
NHI implementation		
Yes	181	55.9
No	130	40.1
Not sure	13	4.0

Almost half of respondents (n = 162, 52.2%) had contracted some form of chronic diseases and the other half came to the clinics for acute illnesses. Among patients with chronic conditions, 123 patients suffered hypertension (75.9%), diabetes n = 79 (48.8%) and IHD n = 15 (9.3%) respectively. A patient may have multiple co-morbidities simultaneously. A total of 43 (26.5%) had two co-morbidities and 6 (3.7%) respondents had three chronic conditions. Among those with acute illness, 61 (37.7%) respondents had upper respiratory tract infections (URTI), 111 (68.5%) had some form of viral fever, and 15 (9.3%) had experienced acute gastroenteritis (AGE) within the last 6 months. Out of 324 respondents, the majority of them (72.2%) had never practiced Traditional and Complementary Medicine (TCM) and the rest admitted practicing some form of TCM.

### Patients’ willingness to pay for their drugs

A total of 234 out of 324 respondents (72.2%) were not willing to pay for any drug charges and 34 (10.5%) of them strongly disagreed on any sort of drugs payment. Only 46 (14.2%) respondents were willing to pay for chronic condition or acute illness drugs, and another 6 (1.9%) were uncertain.

Respondents’ health condition was not significantly associated with their WTP for drugs (p = 0.356). Only 28 (17.3%) out of 162 respondents with chronic condition were found to be willing to pay for their drugs, while for respondents with acute illness, only 22 (13.6%) were willing to pay. Among respondents with hypertension, 19.5% were willing to pay for their drugs followed by respondents with diabetes (13.9%) and with IHD (6.7%).

Among respondents who have two co-morbidities, 14% were willing to pay for their drugs, while 16.7% with three co-morbidities were willing to pay for their drugs. However, co-morbidities were also not significantly associated with patients’ WTP.

The overall level of WTP for drugs are presented using the median value and its’ interquartile range (IQR). Patients were willing to pay for chronic diseases treatment at MYR10 (IQR 1, 30) and slightly higher range was seen at MYR10 (IQR 5, 30) for acute illnesses. However, the Mann–Whitney-U Test showed that there was no significant difference between patients’ WTP for either chronic or acute illness (p = 0.588).

Among diabetic patient, the median value (MYR) and IQR to pay for diabetic drugs was at MYR 10 (1, 25); hypertension at MYR10 (1, 30) and IHD at MYR1 (0, 40). Among patients with acute illnesses i.e. URTI, viral fever and AGE, levels patients were willing to pay for drugs at the median value of MYR10 (IQR 5, 25), MYR10 (IQR 5, 30) and MYR10 (IQR 1, 45) respectively.

A total of 181 respondents (55.9%) agreed to the suggestion that the government is right to implement a national health insurance scheme in the near future. 98 respondents of the 181 respondents (54.1%) chosen to pay the premium at only 1% from their monthly basic salary.

### Factors associated with patients’ WTP for their drugs

Table 2 shows the bivariate analysis elucidating the association between socio-demographic factors and patients’ WTP for drugs. Amongst all socio-demographic factors, personal income and household income were found to have significant relationship with patients’ WTP (p = 0.028 and p = 0.022 respectively). One of the inclusion criteria for respondents, was patients must be 18 years old and above. Age was initially stratified into 4 categories. They were less than 30 years old (i.e. 18 to ≤30 years old), 30–40 years old, 40–50 years old and more than 50 years old. Then we tested for normality and calculated the median age of patients. The relationship between the above 4 age categories and WTP (2 categories of Yes and No) was then analysed. The Chi square analysis was not significant. Then we tested between actual value of age and amount WTP using Spearman correlation. This was also not significant. The median value was 47 years old. Lastly using the Chi square analysis, age is classified into two categories which were ‘younger and older’ age. The group 18–47 was classified as “younger” and 48 and above is classified as “older” age group. The two categories were then tested against WTP. This was also not significant. The final test through multivariate analysis also did not show any significant findings. Perhaps even though the age here varies but the purchasing power of the patients are almost similar. Hence did not show any relationship between age and WTP, unlike income of patients. Higher level income group was more willing to pay for their drugs compared to the low income group, at 19.9% compared to 11.0%. Respondents with higher income level were more willing to pay for drugs (20.1%), compared to the lower level income group (10.9%). Among respondents with chronic conditions, household income was significantly associated with patients’ WTP. Among the respondents suffering from hypertension, 32.5% of the higher income group was willing to pay, compared to respondents with diabetes (23.8%) and IHD (30.0%). This relationship was significant at p = 0.012.

**Table 2:** The association between socio-demographic factors and willingness to pay for drugs (n = 324)

Variables	WTP for drugs		χ^2^	p value
	Yes (%)	No (%)		
Age (years)				
18–47	21 (12.4)	148 (87.6)	2.446	0.118
48 and above	29 (18.7)	126 (81.3)		
Gender				
Male	25 (17.1)	121 (82.9)	0.582	0.445
Female	25 (14.0)	153 (86.0)		
Ethnicity				
Malay	29 (15.1)	163 (84.9)	0.039	0.844
Non-Malay	21 (15.9)	111 (84.1)		
Marital status				
Married	43 (16.8)	213 (83.2)	1.741	0.187
Single	7 (10.3)	61 (89.7)		
Level of education				
Lower level of education	36 (14.6)	210 (85.4)	0.499	0.480
Higher level of education	14 (17.9)	64 (82.1)		
Personal income				
Lower level (RM0-950)	18 (11.0)	145 (89.0)	4.842	0.028*
Higher level (RM951 and above)	32 (19.9)	129 (80.1)		
Household income				
Lower level (RM0-1900)	18 (10.9)	147 (89.1)	5.271	0.022*
Higher level (RM1901 and above)	32 (20.1)	127 (79.9)		
Number of children				
Less than 3	37 (19.1)	157 (80.9)	4.909	0.027*
4 and above	13 (10.0)	117 (90.0)	1.051	
Status of prior insurance				
Have insurance	19 (18.4)	84 (81.6)		0.305
No insurance	31 (14.0)	190 (86.0)		

*WTP willingness to pay*

* *p* < *0.05; χ* ^*2*^ *Pearson Chi Square*

The number of dependents’ children have a significant association with patients’ WTP (p = 0.027). Respondents having between 0 and 3 children were more willing to pay for drugs (19.1%) compared to those who had 4 and above dependents (10.0%). However, stratification analysis based on chronic conditions showed that there was no significant association between numbers of dependent children with patients’ WTP. Other factors such as age, gender, ethnicity, marital status, having prior insurance status and level of education were found to have no significant association with patients’ WTP for drugs.

Further analysis via multivariate logistic regression (Table 3), the Wald’s estimates gave the upmost importance to number of dependents towards patients WTP (p = 0.009). Respondents with lower number of dependents (0 to 3 children) were nearly three times more likely to pay for drugs, compared to those with higher dependents (≥4 children) at adjusted OR = 2.63 (95% CI 1.27, 5.44).

**Table 3:** Logistic regression of influencing factors towards willingness to pay

Variable	β	S.E	Wald	p value	Exp (β)	95% CI for exp (β)	
						Lower	Upper
Age							
Older	0.428	0.441	0.941	0.332	1.534	0.646	3.642
Younger					1.000		
Gender							
Male	0.037	0.342	0.012	0.913	1.038	0.531	2.031
Female					1.000		
Ethnicity							
Non-Malay	0.047	0.337	0.019	0.889	1.048	0.542	2.027
Malay					1.000		
Marital status							
Married	0.576	0.483	1.419	0.234	1.778	0.690	4.585
Single					1.000		
Level of education							
Higher level	0.128	0.425	0.091	0.763	1.137	0.494	2.616
Lower level					1.000		
Personal income							
Higher level	0.449	0.477	0.887	0.346	1.567	0.615	3.988
Lower level					1.000		
Household income							
Higher level	0.510	0.403	1.600	0.206	1.666	0.755	3.673
Lower level					1.000		
Type of occupation							
Employed	0.092	0.454	0.041	0.840	1.096	0.450	2.671
Unemployed					1.000		
Number of dependents							
0 to 3	0.965	0.372	6.732	0.009**	2.625	1.266	5.442
4 and above					1.000		
Type of health condition							
Chronic	0.033	0.406	0.007	0.935	1.034	0.467	2.290
Acute					1.000		
TCM practice							
Practice TCM	0.045	0.375	0.014	0.905	1.046	0.502	2.179
Not practice TCM					1.000		
Health insurance							
No health insurance	−0.127	0.382	0.110	0.740	0.881	0.416	1.864
With health insurance					1.000		

*β* standardized coefficient, *S.E* standard error, *Exp (β)* odds ratio

** p < *0.05*

## Discussion

The patients’ profile showed that the majority of patients who attended the facilities were from the younger age group 18–47 years, females, of Malay ethnicity, completed lower education, lower income group and suffered from chronic condition. This fits with the scenario of urbanised population in Malaysia that attends the subsidised free primary care. Almost 30% of the 162 patients with chronic condition attendees had more than one chronic condition, and hypertensive disease predominates. As in many upper developing countries, unhealthy diet, sedentary lifestyle and chronic diseases such as hypertension are a one of the major issue in this country as well. Among the acute illnesses, the majority of patients contracted acute fever within the last 6 months.

As high as 72.2% of respondents were not willing to pay for drugs. However, 55.9% of respondents supported the future implementation of the proposed future National Health Insurance to be implemented by the government [5]. According to data published [15] by the Department of Statistics Malaysia in 2010; a household average monthly expenditure, consisting of an average of four members per household was approximately MYR 2200. This government-funded survey revealed that the average Malaysians spend 23% of their household income on housing and utilities, 20% for foods and beverages and only 1% is spent for health [15]. This shows that on average, a household is willing to spend approximately MYR 22 per month on health. This amount reflected the tendency of population to be heavily dependent on the long standing universal health coverage provided and minimal cross subsidisation. The mean income per capita for Malaysian was at MYR 5000 per month in the year 2012. Inequality exists with urbanites progressing further than ruralises. The urban household monthly income had increased at a rate of 6.6% per year. This was from MYR 4705 monthly in 2009, to MYR 5742 last 2015 while the rural household monthly income increased 6.4% annually from MYR 2545 to MYR 3080. This leads to the rural population at higher risk due to higher financial commitment of household dependents, children’s education, housing rent, transport (cars, motorbike) instalment and other responsibilities [1, 5]. From Table 2, females, Malay and singles, were less willing to pay for drugs. However these associations were not significant.

Almost all clinics attendees pay a nominal fee of MYR 1 (or USD 0.38), including for all investigations and drugs prescribed per visit [1–3]. Respondents also recommended that the government should continuously subsidies the health care services especially for lower income group earners. Patients with chronic conditions have to undergo a considerable amount of suffering compared to those with acute or common illnesses [7–10]. In addition, it is obligatory for them to go to the clinic more often for follow up and continuation of treatment [1, 2]. If drugs charges are to be implemented abruptly in health clinics without proper targeting for fee subsidisation, this situation may decrease visits to hospitals or clinics in the future [4, 5, 7, 10]. However, our statistical test proved that health status has no significant association with patients’ WTP for drugs. Regardless of a primary care patient’s health condition of having chronic or acute illness, this did not influence the WTP for drugs [8, 16]. Most patients were not enthusiastic to pay for their drugs and proposed that the government drug subsidy to be sustained [1, 5, 14]. The economic effect of chronic disease has adversely and disproportionately affected poor and vulnerable populations in the developing world [13, 16] including Malaysia. The results indicate that only 17.3% of respondents with chronic condition are willing to pay for their drugs. Respondents with hypertension have the highest percentage of WTP as compared to diabetes and IHD. This is in tandem with Bradford et al. finding, which state that hypertensive patients are more responsive to pay compared to other patients’ with other chronic conditions [16, 17].

Our findings illustrated that personal and household incomes were significantly associated with WTP for drugs, for both chronic and acute illness. Those who earned higher income were more inclined to pay more for drugs. This result supports our hypothesis and was consistent with the previous studies, which indicated that the higher income level with more disposable income is associated with WTP [18, 19, 21]. This relevancy of income and WTP, indicate that if drug charge is to be implemented in public dispensing, this may potentially become a burden to the countries of lower income groups. Interestingly even though patients with higher income were more enthusiastic to pay more for drugs, the percentage was very low. The current price inflation and exuberant cost of living have led to the drastic increase in the cost of drugs and healthcare as a whole [18, 20, 21], albeit a higher personal or household income [10, 23].

Respondents with less number of dependents were more willing to pay for drugs (19.1%). This finding is consistent with previous study by Okyere which found that the chances of an individual to pay for a ¢5000 health insurance premium, would be reduced by 10.8%, if there is an increase of 1% in the dependency ratio in the family [18, 19]. This elucidated that an increase in number of dependents was associated with reduced WTP. The concept of opportunity cost is seen here [1, 4]; if patients are required to pay more for expensive chronic disease drugs, this would affect their other daily expenses and basic needs [23]. Hence, this substantiates our hypothesis that patients with a high number of dependents have low WTP for drugs as compared with those with low number of dependents. Therefore, the increase in the number of dependents will contribute to less willingness to pay.

The median value of WTP for drugs among patients who have two co-morbidities was MYR 5 per visit. Whereas for patients who have three co-morbidities were willing to pay up to a higher level at MYR 10.50 but this was not statistically significant. Valderas [20] stated as the number of chronic disease increases, the costs of the treatment will also escalate [3, 22]. However from Chang states those co-morbidities had no significant increase towards patients’ WTP [21]. WTP has its inert weakness [1, 6] i.e. the population with more disposable income would generally be more willing to pay for drugs [9, 12] compared with population who have lower purchasing power and income [5]. Bivariate analysis statistically showed that patients who earned less than MYR950 per month (considered to be lower income) are less likely to pay (89%) for drugs compared to those who earned more than MYR950 per month (80.1%).

The median value patients are willing to pay for drugs was only MYR 10. Taking the highly prevalent diabetes as an example [15, 16, 22] the first line medication prescribed to patient after adequate practice on healthy diet and regular exercise is bi-guanides (Metformin) [22]. For a patient who consumes the maximum dose of Metformin (1000 mg two times daily), he or she requires 120 tablet per month which costs about MYR 15 to MYR 30 [22, 23]. This rough estimation indicates that most Malaysians are not prepared to endure [3, 5] the entire cost of their chronic treatment.

Patients who were not practicing TCM were more willing to pay for drugs as compared with those currently practicing TCM. However, the association of WTP and TCM practice were not significant. Previous studies revealed that patients who contracted chronic condition are more likely to utilize and purchase complementary and alternative medicine [24–26]. They may perceive less need on modern drugs [26], or need to purchase them; hence the less willing to pay.

### Limitation of study

Since this study did not represent the private sector primary care patients, the result would be very centered towards public primary care patients. Thus, it is suggested that future study should include private primary care. As this state is also one of the richest urbanized states in Malaysia, population of WTP would be expected to greatly contradict from other less developed states with a larger number of low income groups.

### Recommendation

The majority of the respondents (72.2%) were not willing to pay for their drugs. Utilisations on other mechanisms need to be addressed tin inspiring its citizens to share the burden of drug cost. It is proposed that public facilities start to implement a staggered payment mechanism whereby step-by-step fee charges are implemented within the next few years period in order to heighten citizens’ awareness in assisting the government to control health care costs. Phasing these implementations would be much palatable to educate the public and finally remove the norm of citizens who currently enjoy the highly subsidized drugs and health care.

Prior implementing the new health care financing policy such as the proposed National Health Insurance and its benefit packages, it is plausible to start charging MYR 10 as the minimum drug charges for each outpatient visit that entails subsidised treatment for acute and chronic diseases. In countries that already implement the casemix costing charges, this can be used to start charging patients according to the cost calculated in the casemix system. Government facilities should start charging the more affluent population on health services and drugs consumed. The minimum value of MYR10 is considering the overall median value of patients’ WTP for drugs (MYR 10 per visit). However proper targeting is needs for the lower income group. This is whilst excluding the vulnerable groups such as the low income earners, pensioners or the elderly. This can be carried out through the implementation of means tested financing.

## Conclusions

The results indicate that patients have low WTP for drugs for both chronic and acute illness even in an urban state of Malaysia. The maximum amount that public patients are willing to pay for acute or chronic diseases drugs are considered low, the median value was only MYR 10 (USD 3.8) per visit. Only three factors were found to be associated with WTP; which are the level personal and household income, and number of dependents. Most of our citizens are in a comfort zone of receiving highly subsidized health services thus, and may not be prepared to transform from the norm of having free services and to pay for his/her own drugs.
